# Improving Clinical Prediction of Bipolar Spectrum Disorders in Youth

**DOI:** 10.3390/jcm3010218

**Published:** 2014-03-10

**Authors:** Thomas W. Frazier, Eric A. Youngstrom, Mary A. Fristad, Christine Demeter, Boris Birmaher, Robert A. Kowatch, L. Eugene Arnold, David Axelson, Mary K. Gill, Sarah M. Horwitz, Robert L. Findling

**Affiliations:** 1 Center for Autism, Cleveland Clinic, Cleveland, OH 44104, USA; 2 Department of Psychology, University of North Carolina at Chapel Hill, Chapel Hill, NC 27599, USA; E-Mail: eay@unc.edu; 3 Department of Psychiatry, Division of Child and Adolescent Psychiatry, Ohio State University, Columbus, OH 43210, USA; E-Mails: mary.fristad@osumc.edu (M.A.F.); l.arnold@osumc.edu (L.E.A.); 4 Department of Psychiatry, Division of Child and Adolescent Psychiatry, University Hospitals Case Medical Center, Cleveland, OH 44106, USA; E-Mail: christine.demeter@uhhospitals.org; 5 Department of Psychiatry, Western Psychiatric Institute and Clinic, University of Pittsburgh Medical Center, University of Pittsburgh, Pittsburgh, PA 15213, USA; E-Mails: birmaherb@upmc.edu (B.B.); axelsonda@upmc.edu (D.A.); gillmk@upmc.edu (M.K.G.); 6 Department of Psychiatry, Nationwide Children’s Hospital, Columbus, OH 4320, USA; E-Mail: robert.kowatch@nationwidechildrens.org; 7 Department of Child and Adolescent Psychiatry, New York University School of Medicine, New York, NY 10016, USA; E-Mail: sarah.horwitz@nyumc.org; 8 Department of Psychiatry, Johns Hopkins University, Baltimore, MD 21287, USA; E-Mail: rfindli1@jhmi.edu

**Keywords:** bipolar disorder, children, risk factors, clinical decision making, classification tree analysis

## Abstract

This report evaluates whether classification tree algorithms (CTA) may improve the identification of individuals at risk for bipolar spectrum disorders (BPSD). Analyses used the Longitudinal Assessment of Manic Symptoms (LAMS) cohort (629 youth, 148 with BPSD and 481 without BPSD). Parent ratings of mania symptoms, stressful life events, parenting stress, and parental history of mania were included as risk factors. Comparable overall accuracy was observed for CTA (75.4%) relative to logistic regression (77.6%). However, CTA showed increased sensitivity (0.28 *vs*. 0.18) at the expense of slightly decreased specificity and positive predictive power. The advantage of CTA algorithms for clinical decision making is demonstrated by the combinations of predictors most useful for altering the probability of BPSD. The 24% sample probability of BPSD was substantially decreased in youth with low screening and baseline parent ratings of mania, negative parental history of mania, and low levels of stressful life events (2%). High screening plus high baseline parent-rated mania nearly doubled the BPSD probability (46%). Future work will benefit from examining additional, powerful predictors, such as alternative data sources (e.g., clinician ratings, neurocognitive test data); these may increase the clinical utility of CTA models further.

## 1. Introduction

Diagnosis of bipolar spectrum disorders (BPSD) in youth remains a difficult but important clinical responsibility. Assessment strategies are needed that are inexpensive, easy to implement, have good predictive power, and simultaneously combine multiple pieces of clinical information to increase or decrease the probability of BPSD. This presents a significant challenge because the optimal statistical methods for combining multi-faceted clinical information, such as logistic regression, are often difficult to implement in typical clinical practice and do not map clearly onto clinical decision making [1].

In a recent paper by Fristad and colleagues [2], logistic regression analyses suggested decreasing incremental value in predicting BPSD using information from parent ratings of mania at screening and baseline, family history of parental mania, and parenting stress. This work suggested an initial assessment strategy that may be clinically useful for screening out individuals with low probability of BPSD and identifying individuals with higher probability of BPSD who would benefit from more detailed assessment. However, several possibilities exist for implementing this assessment strategy. The simplest approach would be to examine results for each significant predictor and implicitly weight their importance. Unfortunately, large volumes of literature argue against this approach to clinical decision making [3,4,5]. For example, decisions based on clinical intuition tend to be inaccurate or highly variable and are extremely sensitive to the base rate of the condition in the setting where the decision is being made [6,7].

More detailed and defensible methods involve explicit weighting of predictors. One weighting approach would be to use the regression weights derived from logistic regression analyses. Although familiar within the research community, logistic regression analyses are difficult to implement in clinical practice. They require generating a complex combination of scores multiplied by regression coefficients to produce post-test probabilities of having BPSD. Additionally, logistic regression estimates are optimized for the study participants from which they are derived and thus, may not replicate well across clinical settings [8]. Logistic regression assumes a linear relationship between predictors (test scores) and the transformed odds of the criterion (diagnosis). In contrast to logistic regression, newer methods, such as classification tree approaches, can accommodate both linear and non-linear relationships. Finally, application of regression weights requires that complete information about all of the variables in the model is available for the new clinical case. For instance, if the regression model includes family history of mood disorder as a predictor, then weights for other variables are adjusted based on inclusion of family history in the model, and the model cannot be used with cases that are missing family history (or any other predictor variable). Clearly, alternative methods are needed that are empirically defensible, easier to implement, and that better approximate clinical decision-making.

The purpose of the present paper is to extend the work of Fristad and colleagues [2] by using classification tree analyses (CTA). CTA is an iterative method that optimizes classification at each step by selecting the strongest classifier variable while simultaneously avoiding over-fitting through the use of bootstrap re-sampling, and providing results in a clinically-useful tree diagram form [9]. CTA-derived algorithms have the potential to produce equally or more accurate overall classification relative to logistic regression, provide a superior balance of sensitivity and positive predictive power (important for identifying low base rate conditions), and provide visual tree diagrams that can be helpful for simplifying the integration of complex clinical information during the decision process (e.g., [10,11]). We anticipate that CTA models will more clearly delineate how parent ratings of mania, parental history of mania, stressful life events, and parenting stress can be integrated to increase or decrease the probability of BPSD—providing concrete recommendations for clinical decision-making.

## 2. Experimental Section

For this study, the same sample, study design, and data collection procedures were used as in the paper by Fristad and colleagues [2]. Institutional Review Boards at each of the four Longitudinal Assessment of Manic Symptoms (LAMS) sites (Case Western Reserve University, Cincinnati Children’s Hospital Medical Center, the Ohio State University, and the University of Pittsburgh Medical Center/Western Psychiatric Institute and Clinic) approved all protocol procedures.

### 2.1. Sample Ascertainment

The source population consisted of all eligible youth aged 6 through 12 years visiting 9 child outpatient mental health clinics (2 in Cleveland, 1 in Cincinnati, 5 in Columbus and 1 in Pittsburgh) associated with universities in the LAMS study. Exclusion criteria included: (1) a prior visit to the same clinic within the preceding year; (2) not being accompanied by a parent or legal guardian; (3) having a parent who did not understand or speak English; and (4) having a sibling or other child living in their household who had already participated in LAMS screening [12].

Caregivers completed the Parent General Behavior Inventory 10-Item Mania Scale (PGBI-10M) [13] to screen for elevated symptoms of mania (ESM). Items comprising the PGBI-10M describe hypomanic, manic, and biphasic symptoms and have been reported to discriminate BPSD in youth from other diagnoses [13]. Items are scored from 0 (“never or hardly ever”) to 3 (“very often or almost constantly”); total scores range from 0 to 30 with higher scores indicating greater symptoms. Each patient whose parent/guardian rated the child ≥12 (ESM+) on the screening PGBI-10M was invited to participate in the longitudinal portion of the LAMS study. A smaller comparison group of patients who scored 11 or lower (ESM−) matched on age, sex, race, ethnicity, and Medicaid status was also selected. Of 1124 children who screened ESM+, 621 or 55% accepted the invitation. Information including age, sex, race, ethnicity, and health insurance status was obtained from parents/guardians. There were no socio-demographic differences between children/families agreeing to enroll in the longitudinal study and those who did not. The mean time interval between screening and baseline assessment was 45.5 (SD = 41.4) days. ESM− children were selected from the available pool to match on age (±2 years), sex, race/ethnicity and insurance status; 86 children without ESM (ESM−) were included in the longitudinal cohort [12]. It was anticipated that some, but not all children who were ESM+ would receive diagnoses of a BPSD upon completion of the baseline assessment described below.

### 2.2. Risk Factors and Clinical Diagnoses

Risk factors eligible to be included in predictive models were: parent reports of mania symptoms at screening and baseline using the PGBI-10M (range 0–30) [13], parent history of mania (PHM; any history present *vs*. absent), Parent Stress Survey (PSS; [14]) scores ranging from 0 (no stress) to 100 (high stress), and Stressful Life Events Schedule (SLES; [15]) scores (range 0–34). The criterion was BPSD diagnosis (BPSD *vs*. no BPSD), collapsing bipolar I, bipolar II, cyclothymic disorder, and bipolar not otherwise specified (NOS) into BPSD and all other diagnoses or no diagnosis into no BPSD. All diagnoses followed strict DSM-IV criteria [16], with NOS following the operational definitions used in the Course and Outcome of Bipolar Youth study [17].

#### 2.2.1. Diagnoses

Children and their guardians were administered the Schedule for Affective Disorders and Schizophrenia for School-Age Children-Present and Lifetime Episode (K-SADS-PL) [18] with additional depression and manic symptom items derived from the Washington University Kiddie Schedule for Affective Disorders (WASH-U K-SADS) [19,20]. Items assessing nonverbal communication, the child’s relationship with others, shared enjoyment, and social-emotional reciprocity according to DSM-IV criteria were added to the KSADS-PL to screen for pervasive developmental disorders (PDDs). The resulting instrument, the K-SADS-PL-W, is a semi-structured interview that assesses current and lifetime psychiatric diagnoses and time course of each illness. In this report, only current diagnoses at baseline assessment are presented.

Unmodified DSM-IV diagnostic criteria were used. Criteria for BP-NOS were clarified to follow those criteria used in the Course and Outcome of Bipolar Youth study (COBY) [21] and were operationalized as follows: (a) elated mood plus two associated symptoms of mania (e.g., grandiosity, decreased need for sleep, pressured speech, racing thoughts, increased goal-directed activity), or irritable mood plus three associated symptoms of mania; (b) change in the participant’s level of functioning (increase or decrease); (c) symptoms must be present for ≥4 h within a 24-h period; and (d) the participant must have had ≥4 such episodes, or a total of four days of the above-noted symptom intensity in his/her lifetime. To prevent post-training rater drift, all interviewers rated taped administrations of the K-SADS-PL-W taped at each site throughout the course of the study. Kappas were: 0.82 for K-SADS-PL-W psychiatric diagnoses in general and 0.93 for bipolar diagnoses in particular. These are within acceptable levels [22].

All diagnoses were reviewed and confirmed by a licensed child psychiatrist or psychologist.

#### 2.2.2. Parent Report of Mania Symptoms

The PGBI-10M, described above, was administered at screening and baseline.

#### 2.2.3. Family History

The Family History Screen (FHS) [23,24] collected information on 15 proxy psychiatric disorders and suicidal behavior in biological parents. In this study, symptoms of DSM-IV-TR defined mania were evaluated. Individuals were considered positive for a parental history of mania (PHM) if one (score = 0.5) or both (score = 1.0) parents endorsed yes for “extreme elated mood” plus 3 supporting symptoms (extreme or non-extreme- more talkative, inflated self-esteem, decreased need for sleep, racing thoughts, distractible, restless, and excessive involvement in pleasurable activities) or yes for “extreme irritability” plus four supporting symptoms. In cases where there were missing data, if enough symptoms were known to meet the above criteria, the parent was scored positively. If the parent could not meet criteria even if missing symptoms were available (e.g., if neither extreme elated mood nor extreme irritable mood was endorsed), the parent was scored negatively. If there was uncertainty regarding whether or not a parent would or would not meet criteria if missing symptoms were known, the parent was scored as unknown and that child was not included in this sample.

#### 2.2.4. Family Environment

The Parent Stress Survey (PSS) [14] was used to assess parental stress related to raising a psychiatrically impaired child. The PSS is a 25-item parent self-report instrument; each item has a yes or no response to document occurrence of the event, followed by a Likert-type scale, with response choices ranging from 0 (not at all stressful) to 4 (very stressful), to measure the severity of stress experienced. The total score is the sum of the 25 Likert-type items and ranges from 0 (no stress) to 100 (high stress). The questionnaire has a coefficient alpha of 0.87 [25].

#### 2.2.5. Stressful Life Events

The Stressful Life Events Schedule (SLES) [15] collected information on occurrence, date of occurrence, duration, and perceived threat of events experienced by the youth. The SLES also allows for the determination of whether an event was dependent on behaviors of the child or adolescent. The SLES has shown good test-retest reliability (κ = 0.68).

### 2.3. Statistical Analyses

CTAs were computed using optimal data analysis methods [9]. CTAs, although less familiar to many clinical researchers than logistic regression, provide several advantages, including: comparable or better accuracy of prediction relative to logistic regression [26,27,28,29]; ability to model both linear and non-linear relationships between the predictor and criterion; inclusion of predictors only in parts of the model where they improve classification [9]; maintenance of experiment-wise Type I error at 0.05 using a generalized multiple comparison correction to minimize over-fitting; use of jackknife leave-one-out bootstrap re-sampling to identify the most stable predictors and attempt to maximize generalizability of the CTA model; and visual output of classification rules—in the form of a decision tree—that can be easily implemented by clinicians [29]. CTA is conceptually related to other types of classification analyses such as Q-ROC, classification and regression tree, bagging, and random forests [1,30].

Classification tree building via univariate optimal data analysis methods is an iterative process involving the following steps: 

(1) Find the optimal cut-point for the strongest predictor by examining all possible predictors. The strongest predictor is identified based upon maximum classification accuracy across jackknife (bootstrap) re-sampling.

(2) Find the optimal cut-point to create branches that maximize classification accuracy for that predictor. For example, screening and baseline PGBI-10M scores may be expected to be the most powerful predictors. For each PGBI-10M variable, a cut-point is identified (e.g., screening PGBI-10M <12 *vs*. ≥12), creating two branches. Each branch includes individuals who are correctly and incorrectly classified as having or not having a BPSD diagnosis. The cut-point maximizes classification accuracy for PGBI-10M scores.

(3) Examine each branch separately to find the next largest predictor that significantly improves classification. For example, for individuals with elevated screening PGBI-10M scores (≥12), elevated baseline PGBI-10M scores may provide the largest increase in classification accuracy. This offers an opportunity for improvement over logistic regression and other methods, in that different variables could be selected to optimize performance in different branches. Gender might be important in refining classification of high scorers on the PGBI-10M, but not relevant to the low scorers, for example.

(4) Identify the optimal cut-point for this next predictor. Each cut-point represents new branches added to the tree.

(5) Repeat this process for individuals with low screening PGBI-10M scores (<12) and each available branch until no additional predictors add significantly to a branch.

(6) When the tree model is completed, “prune” branches using a Bonferroni adjustment of Type I error to minimize overfitting the tree model.

(7) Compute and visually display the overall predictive performance and classification accuracy of the final tree so clinicians can use this information in decision making. At the end of each branch, the proportions of individuals with and without BPSD are provided. These proportions represent posterior (post-test) probabilities because the relevant risk factors for BPSD have been considered. Posterior probabilities can be compared to the anterior probability (pre-test probability or sample base rate) to evaluate the change in identification of BPSD.

In the present study, CTA results were compared to those obtained using hierarchical logistic regression [2]. Sensitivity, Specificity, Positive (PPV) and Negative Predictive Values (NPV) were computed to compare classification accuracy across methods.

## 3. Results

Table 1 presents the LAMS sample description, separately for children with a baseline BPSD diagnosis and children without a baseline BPSD diagnosis. Children with BPSD were older and more likely to be female than the rest of the sample (albeit still only 42% female). They were less likely to be diagnosed with a disruptive behavior disorder and were slightly, albeit non-significantly, less likely to be diagnosed with an autism spectrum disorder. There were no other differences in demographics or baseline diagnoses. Given that previous analyses of these data [2] found only minor effects of demographic factors, demographic factors were not included in CTA and logistic regression model comparisons.

**Table 1:** Longitudinal Assessment of Manic Symptoms (LAMS) sample description, separately for children with and without bipolar spectrum disorders (BPSD).

Demographic and Clinical Descriptives	BPSD *n* = 148	No BPSD *n* = 481	*t*/*Χ*^2^ (*p*)
**Mean age (SD) at screening**	9.67 (2.10)	9.04 (1.86)	3.28 (0.001)
**Sex (% male)**	58.1	72.3	10.73 (0.001)
**Race (%)**			
White	70.3	63.2	
African-American	20.9	28.3	
Multi-Racial or Other Race	8.8	8.5	
**Ethnicity (% Hispanic)**	2.7	4.2	0.65 (0.419)
**Insurance Status (%)**			2.88 (0.411)
Medicaid	48.0	53.8	
Private	42.6	39.5	
Medicaid and Private	6.8	5.4	
Self-Pay	2.7	1.2	
**Diagnoses (*n*, %)**			
Any attention deficit hyperactivity disorder	106 (71.6)	371 (77.1)	1.88 (0.171)
Any disruptive behavior disorder	63 (42.6)	263 (54.7)	6.65 (0.010)
Any anxiety disorder	48 (32.4)	146 (30.4)	0.23 (0.632)
Any depressive spectrum disorder	0 (0)	104 (21.6)	38.34 (<0.001)
Any psychotic disorder	3 (2.0)	13 (2.7)	0.21 (0.648)
Any autism spectrum disorder	5 (3.4)	35 (7.3)	2.89 (0.089)

**Figure 1:**
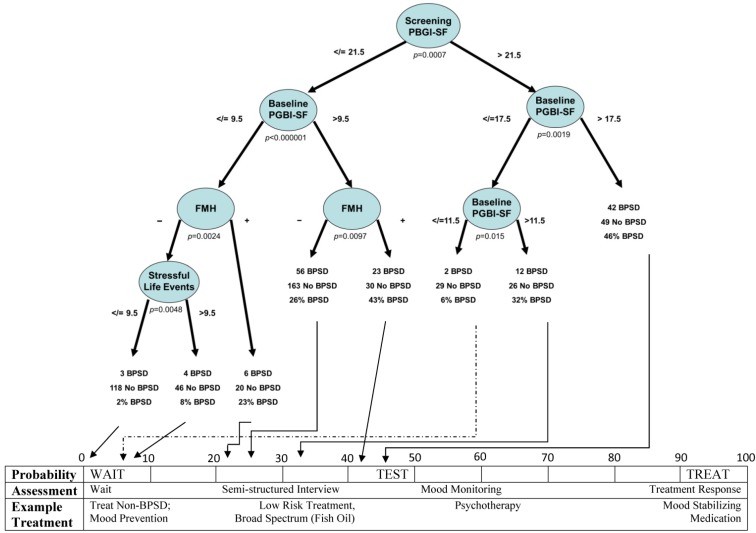
Classification tree analysis decision tree predicting the presence *vs*. absence of a Bipolar Spectrum Disorder (BPSD).

Note: PHM = parent history of mania scored as any history present or absent; PGBI-10M = Parent General Behavior Inventory—10 item mania form. Scores on the PGBI-10M infrequently yielded non-integer values if a parent selected two Likert scale responses (ex. scores of 2 and 3 circled). For this reason, we present cut scores as defined empirically with non-integer values. Assessment and treatment examples in the above figure are NOT evidence-based recommendations but rather represent possibilities that MIGHT map to different levels of the post-test probability continuum if the evidence-base was clear. Other examples of potential clinical actions exist, such as withholding, or using with caution, antidepressants or stimulants for youth with moderate risk of BPSD.

Figure 1 presents the CTA decision-tree diagram with all significant predictors. Both screening and baseline PGBI-10M were significant in the first iteration (*p* < 0.001). Because screening PGBI-10M is collected first, it was entered as the first branch point. Interestingly, PHM entered as a predictor for low screening PGBI-10M scores, but not for high scores. PSS scores did not enter in any branch, suggesting it may account only for variance in the BPSD diagnosis that was already accounted for by PGBI-10M scores and PHM. SLES scores improved prediction only for individuals with the lowest BPSD probability (low screening and baseline PGBI-10M scores, negative PHM).

Several classification tree branches appear to be helpful in increasing or decreasing the probability of BPSD. For example, the 24% sample probability of BPSD was substantially decreased in youth with low screening and baseline PGBI-10M scores, negative PHM, and low levels of stressful life events (2%); for those with low screening and baseline PGBI-10M scores, negative PHM, and high stressful life events (8%); and for those with high screening but fairly low baseline PGBI-10M scores (6%). These branches represent a substantial proportion of the sample (Total *n* = 202; No BPSD = 193, BPSD = 9) and provide excellent negative predictive power (0.96). High screening coupled with high baseline PGBI-10M scores nearly doubled the probability of BPSD (from 24% to 46%). Similarly, a low screening score followed by a high baseline score and positive PHM almost doubled the probability (43%). However, in both cases the probability was still below 50%. It is also crucial to note that all of the branches on the tree are clinically useful for detection of BPSD. For example, considering only increased risk branches (rightward branches), after the first step (screening PGBI-10M) only 38% of BPSD cases are identified as increased risk.

Table 2 presents a comparison of diagnostic efficiency statistics between logistic regression and classification tree analyses. Logistic regression and CTA produced comparable overall accuracy (77.6% *vs*. 75.4%, respectively). However, unlike logistic regression, classification tree analyses (CTA) strike a balance between sensitivity and positive predictive value (PPV) while maximizing weighted accuracy and accounting for the base rate of BPSD. Relative to logistic regression, CTA increased sensitivity (+0.10) while decreasing specificity (−0.06). The balance of positive and negative predictive power was better for logistic regression than CTA due to the restrictive fashion in which accuracy was computed for CTA (all high scores on PGBI-10M required for predicting BPSD).

**Table 2:** Diagnostic efficiency statistics for logistic regression and classification tree analyses (CTA) (*N* = 621).

Diagnostic Efficiency Statistic	Logistic Regression	CTA
Accuracy	0.78	0.75
Sensitivity	0.18	0.28
Specificity	0.96	0.90
Positive Predictive Value (PPV)	0.57	0.46
Negative Predictive Value (NPV)	0.79	0.80
PPV (2+ signs)	-	0.46
NPV (3− or 4− signs)	-	0.96

Note: For CTA, overall diagnostic efficiency statistics were computed with bipolar spectrum disorders (BPSD) as the target when all branches were positive (high PGBI-10M scores, positive PHM); otherwise no BPSD was the target. PPV (2+ signs) was computed as the proportion of BPSD cases when high scores for baseline PGBI-10M and positive PHM were present. NPV (3− or 4− signs) was computed as the proportion of non-BPSD cases when at least 3 negative branches (low PGBI-10M and SLES scores, negative family history).

The pragmatic advantage of CTA becomes clear when combining the last two rows of Table 1 with inspection of Figure 1. CTA permits examination of the most useful combinations of predictors and cut scores for decreasing and increasing the probability of BPSD. For example, screening and baseline PGBI-10M scores, negative PHM, and low SLES scores are very helpful for “ruling out” bipolar disorder (only 3 BPSD cases out of 121 total youth) in a high base rate clinical setting such as the present sample (24% BPSD). CTA is also helpful for showing that several conflicting combinations of predictors (e.g., low screening and baseline PGBI-10M scores with a positive PHM) are not helpful for altering the probability of BPSD. 

## 4. Discussion

The present study demonstrated the utility of CTA in combining the available clinical information to inform a comprehensive assessment. Relative to logistic regression, which requires the cumbersome application of a linear formula, including calculation of the log odds, CTA provides a diagram that can be easily implemented by clinicians. This diagram offers clear information about the positive and negative predictive value of particular combinations of predictors, including both continuous (PGBI-10M, PSS, SLES) and categorical (PHM) predictors. Clinical use of CTA diagrams are also vastly superior to alternative approaches based on mental combinations of predictors or using odds ratios or relative risks in an iterative fashion. This is because CTA explicitly accounts for the shared variance among significant predictors, whereas an iterative approach is prone to over-or under-estimating the probability of BPSD. Furthermore, because CTA classifications are derived using a jack-knife leave-one-out procedure, previous applications have suggested that they may be more likely to generalize to other settings than sample-dependent logistic regression estimates [26,28]. The approach is also more accurate than any single-gate screening using any of the available predictors.

The CTA approach navigates a huge number of possible combinations of scores—more than 9,800,000 in this example based on permutations of the five raw score inputs, or 32 combinations if each were dichotomized and treated as high risk *versus* low risk. CTA combines these predictors and produces estimates that would be impossible to duplicate intuitively, and would be tedious to calculate manually. For example, two low PGBI-10M scores can over-ride even extremely high levels of stress to indicate a low probability of BPSD (<10%), but they are cancelled out by positive family history of mania (23% probability of BPSD, similar to the starting base rate). Conversely, high scores on both the screen and first visit PGBI-10M double the risk of BPSD, and they obviate the need to consider stressful life events with regard to the diagnosis. The potential information conveyed by stressful life events is statistically redundant with the information captured by the elevated PGBI-10M with regard to BPSD diagnoses. This approach also lends to generating even simpler rules of thumb for clinicians. For example, if the second (baseline) PGBI-10M score is >11.5, then the probability of BPSD is at least 26%, a level that would suggest the need for more detailed evaluation.

CTA analyses can readily be combined with the decision-threshold model advocated by Evidence Based Medicine [31,32]. In this framework, the clinician estimates the probability of having a BPSD along a continuum from nearly 0% to nearly 100%. There are two major decision-making thresholds along the continuum, marking changes in the next action that the clinician takes with the patient. One is the Wait-Test Threshold. When the probability falls below this threshold, then the diagnosis is considered “ruled out”—sufficiently unlikely to not require further evaluation, let alone active treatment. Probabilities above this threshold indicate more intensive evaluation, until new information either reduces the probability of BPSD to fall below the Wait-Test Threshold, or else increases the probability to the point that it crosses the second threshold, the Test-Treat Threshold. At that point, active treatment of BPSD would likely be indicated. EBM authorities point out that the position of these thresholds can vary depending on the risks and benefits attached to testing and treatment. It also is possible to map these thresholds onto different “doses” of assessment and treatment [33,34,35].

Connecting the CTA results with the EBM threshold approach shows that the CTA can quickly sort the clinical information to divide cases into “low risk” or “wait zone” *versus* middling risk “test more” cases. This is accomplished using a brief, public domain screen, then augmenting with family history or a stress inventory to reduce false negatives. Cases in the low risk zone all met criteria for at least one Axis I disorder. Appropriate treatments for these other conditions could start with increased confidence that they would not exacerbate an unrecognized bipolar disorder. The other cases, falling in the “Test Zone”, would be appropriate for more intensive evaluation before beginning acute treatment. Options here would include referral to a psychiatrist or specialist, conducting a semi-structured diagnostic interview to systematically explore possible mood disorder, and/or initiation of intensive retrospective or prospective mood monitoring. Semi-structured interviews are likely to be particularly helpful given the low reliability of typical clinical interviewing with regard to bipolar disorder [5,36]. Despite clinician reservations, semi-structured approaches are well-tolerated by patients [37]. These cases also might warrant initiation of psychotherapy focused on improving mood regulation [38], enhancing family communication [39], or coaching about mood and behavior management strategies [40]. Each of these are likely to be helpful if there proves to be a BPSD involved, and each is more likely to help than harm if instead there is “only” unipolar depression, attention-deficit/hyperactivity disorder, a disruptive behavior disorder, or some similar clinical issue.

As an example of a possible clinical application, a parent may be sent a PGBI-10M to be completed prior to their child’s initial appointment. Subsequently, the parent may be given the PGBI-10M and the SLES to be completed at the appointment. Based on findings from these measures, elevated PGBI-SF scores at screening (>21.5) and baseline (>17.5) virtually doubles the probability of BPSD from the sample base rate of 24% to a posterior probability of 46% (*i.e*., probability after accounting for the risk factors).

In a world of decreasing resources and increasing managed care, this would aid in prioritizing who should receive further evaluation for BPSD. Alternatively, a patient with low scores on the PGBI-10M at screening (≤21.5) and baseline (≤9.5), coupled with a negative family history in the biological parents and a low score on the SLES (≤9.5), decreases probability of BPSD almost 12-fold (0.02 posterior probability). The CTA approach identifies cases for more intensive follow-up, and this may provide sufficient “medical necessity” to facilitate reimbursement by third party payors.

Prior to widespread implementation, the CTA results from the present study will require replication and extension to other clinical and community samples. Analyses should also proceed in larger samples where CTA analyses can be re-computed in specific demographic sub-samples. This will be particularly important given that Fristad and colleagues [2] identified small but potentially important differences in the value of predictors across demographics. Fortunately, if replicated, adoption in typical clinical practice is likely to yield a favorable trade-off between a slight increase in evaluation time (consulting the CTA diagram) and a substantial increase in the accuracy of clinical decision making.

The primary limitations of the present study were the lack of follow-up information on BPSD and the modest number of predictors evaluated. Additional waves of LAMS data collection will be useful for extending the present analyses to evaluate both prediction of all BPSD cases and prediction of new onset BPSD cases. The number of predictors was limited by the available information at baseline, but was also chosen for comparability to the logistic regression analysis results of Fristad and colleagues [2]. Future work would benefit from examining additional enhancements to the CTA approach, such as generating PGBI-10M symptom measurements via computerized adaptive testing implementing item response theory, rather than static paper-pencil questionnaires. Additional research is also needed examining other behavioral, neurocognitive, or biological potential predictors of BPSD in youth.

## 5. Conclusions

The present study demonstrates the utility of CTA in conjunction with brief, readily attained assessments in modifying the probability of a BPSD diagnosis. In the present context, CTA had greater utility and is more clinically useful than logistic regression. Future work will benefit from examining additional, powerful predictors, such as clinician ratings, neurocognitive test data, and/or structural and functional neuroimaging patterns. Addition of more powerful predictors is likely to further enhance the accuracy, clinical utility, and caregiver acceptability of the diagnostic process. This work should also consider the developmental course and development of BPSD, a crucial next step for analyses of the LAMS sample.
